# Treatment received, satisfaction with health care services, and psychiatric symptoms 3 months after hospitalization for self-poisoning

**DOI:** 10.1186/1744-859X-11-10

**Published:** 2012-04-20

**Authors:** Tine K Grimholt, Mari A Bjornaas, Dag Jacobsen, Gudrun Dieserud, Oivind Ekeberg

**Affiliations:** 1 Department of Acute Medicine, Oslo University Hospital Ulleval HF, Oslo, Norway; 2 Norwegian Institute of Public Health, Oslo, Norway

**Keywords:** Follow up, depression, psychiatric symptoms, self-poisoning, suicide attempt, treatment, satisfaction

## Abstract

**Background:**

Patients who self-poison have high repetition and high mortality rates. Therefore, appropriate follow-up is important. The aims of the present work were to study treatment received, satisfaction with health care services, and psychiatric symptoms after hospitalization for self-poisoning.

**Methods:**

A cohort of patients who self-poisoned (n = 867) over a period of 1 year received a questionnaire 3 months after discharge. The Beck Depression Inventory (BDI), Beck Hopelessness Scale (BHS), and Generalized Self-Efficacy Scale (GSE) were used. The participation rate was 28% (n = 242); mean age, 41 years; 66% females.

**Results:**

Although only 14% of patients were registered without follow-up referrals at discharge, 41% reported no such measures. Overall, satisfaction with treatment was fairly good, although 29% of patients waited more than 3 weeks for their first appointment. A total of 22% reported repeated self-poisoning and 17% cutting. The mean BDI and BHS scores were 23.3 and 10.1, respectively (both moderate to severe). The GSE score was 25.2. BDI score was 25.6 among patients with suicide attempts, 24.9 for appeals, and 20.1 for substance-use-related poisonings.

**Conclusions:**

Despite plans for follow-up, many patients reported that they did not receive any. The reported frequency of psychiatric symptoms and self-harm behavior indicate that a more active follow-up is needed.

## Introduction

Self-poisoning is a common cause of hospitalization and is associated with complex and serious health problems [1]. Compared with the general population, the suicide risk is higher in self-poisoning patients [2,3]. Mental illness, alcohol and substance misuse, social isolation, and physical health problems increase suicide risk [1]. Further, depression and anxiety are predictors of premature death among drug abusers [4]. Mortality from natural and unnatural causes is significantly increased among these patients compared with the general population [5]. Furthermore, in a previous study from Oslo, nearly 30% of all acute patients hospitalized for poisoning repeated the poisoning during the first year after the index episode [6].

The main reasons for self-poisoning are suicidal behavior or related to substance misuse. The intention, however, can be difficult to assess and may vary among different episodes of hospitalization for self-poisoning in the same patient [6]. The clinical evaluation of intention will influence the type of follow-up that is arranged before discharge. Accordingly, the motivation for follow-up may vary. Substance misusers that are hospitalized because of accidental poisoning may be less motivated than suicide attempters. The motivation may also be related to the level of depression, hopelessness, and self-efficacy.

Psychiatric symptoms, especially depression and feelings of hopelessness, are well known contributive factors to an episode of self-poisoning; however, there is little knowledge about the incidence of these symptoms after discharge in a total population with self-poisoning including both substance-related and suicidal-related poisonings. The acute hospital stay is often short [7], which makes it difficult to solve complex problems. Although a large proportion of patients with self-poisoning pass through the emergency department, not all patients get a psychosocial assessment [8] or further contact with mental health services [9]. Active follow-up and intervention shortly after discharge, together with adequate treatment strategies, can be preventive [10]. Knowledge about adherence to, and satisfaction with, health care services can help develop and provide enhanced services to this patient group [11].

Therefore, our aims were to study (1) the kind of treatment the patients had received 3 months after hospitalization for self-poisoning and whether this varied according to the intention evaluated, (2) satisfaction with care during the initial hospital stay and the follow-up period, (3) if the patients had engaged in repeated acts of self-harm, (4) how the patients perceived their need for professional help, and (5) their level of depression, hopelessness, and generalized self-efficacy.

## Methods

In this prospective cohort study, all patients that were hospitalized for self-poisoning in Oslo and in the neighboring municipality of Bærum over a period of 1 year were followed-up 3 months after discharge. The initial inclusion of all acute cases of poisoning was performed consecutively from 1 April 2003 to 31 March 2004. The clinical and epidemiological data from Oslo was reported previously [7,12,13]. The present study included self-poisoning exclusively. All cases that were considered as accidental poisonings, such as carbon monoxide poisoning caused by fire accidents, or ingestion of prescribed medication in incorrect doses because of a lack of understanding the prescription were excluded. In addition, patients with no fixed abode, those who died before the 3-month follow-up, or those with unknown identity were excluded. Patients that were admitted more than once during the study period received a new postal form if it was more than 1 month since their last hospitalization.

Finally, 867 patients were eligible for the present study and received a postal self-report form 3 months after discharge. After 3 weeks they also received a reminder. The response rate was 41%. Among the 360 questionnaires returned, 115 did not want to fill out the questionnaire and 3 were incomplete; finally, 28% (242) questionnaires were included in the final analyses.

### Hospital registrations

In the hospital, the treating physician obtained data by completing a standardized registration form as soon as the patient was ready for an interview. The variables used in the present study were evaluated intention, sociodemographics, previous or current psychiatric treatment, previous suicide attempts, and self-reported use of substances. Referrals to follow-up services at the time of discharge were also recorded, and more than one category was recorded for each patient. Some patients left the hospital against medical advice and were treated as a separate group in further analyses.

Evaluation of intention was based on all information available, including the patients' own reports, and was classified in a registration form. Three categories were used: suicide attempt (possible or definite), appeal, and substance-use-related poisoning. Patients who had attempted suicide included those evaluated by the treating physician as having a moderate to high suicidal intent. Appeal patients were those with low or no suicide intent; for example, those who took a dose that they knew was not lethal or took a minor dose with other people present. In these cases, self-poisoning could not be classified as a substance-use-related poisoning or as a suicide attempt, as suicidal intent was low or non-existent. Patients with substance-use-related poisoning had misused substances (ethanol, opiates or opioids, γ-hydroxybutyrate (GHB), amphetamines, ecstasy, cocaine, benzodiazepines, cannabis, or a combination of substances) in a way that led to hospitalization; in these cases, the intended purpose was thought to be recreational use. The distinction between the three categories was not necessarily clear cut, but the physicians were asked to categorize each patient into one of the groups based on their best clinical judgment. To separate suicide attempts from appeals, special attention was given to letters that confirmed suicidal intent, supposed lethal doses of the toxic agent, or other measures taken to ensure a lethal outcome. Information from other sources, such as ambulance personnel and companions, was also considered.

### Treatment received and satisfaction with care

To compare the kinds of treatment offered to the patients before discharge from the hospital with those they actually received, and assess the time from discharge until the first follow-up appointment, we developed a questionnaire aimed at measuring whether or not patients had received the different treatment options that had been registered.

Further questions regarding satisfaction with treatment during the hospital stay and different health care services were added. They were scored on a five-point Likert scale, from 1 (very dissatisfied) to 5 (very satisfied).

### Psychiatric assessments at follow-up

The patients were asked to report repeated episodes of intentional self-poisoning using pharmaceuticals, cutting, or other potentially harmful injuries (not specified) in the 3-month period after hospital discharge. They also assessed their self-perceived psychiatric and substance misuse problems (anxiety, depression, pain, eating disorders, hallucinations, paranoid ideation, alcohol and substance misuse, and need of help) using a five-point scale ranging from 1 (no problems at all) to 5 (very serious problems, need to be hospitalized). Furthermore, the following instruments were used: the Beck Depression Inventory (BDI) [14], the Beck Hopelessness Scale (BHS) [14], and the Generalized Self-Efficacy Scale (GSE) [15].

The BDI measures the severity of depression during the previous week. It is composed of 21 items related to depressive symptoms. Each item has a set of at least four possible answers, varying in intensity. The standard cut-offs are: scores of 0 to 9 indicate that a person is not depressed, 10 to 18 indicates mild to moderate depression, 19 to 29 indicates moderate to severe depression, and 30 to 63 indicates severe depression [14].

The BHS [14] is a 20-item scale with true/false statements for measuring positive and negative expectations about the future. The total BHS score ranges from 0 (no hopelessness) to 20 (maximum hopelessness). The classification of scores is: 0 to 3, minimal; 4 to 8, mild; 9 to 14, moderate; and 15 to 20, severe hopelessness [16].

The GSE comprises ten items assessing the strength of an individual's belief in his/her ability to respond to novel or difficult situations and to deal with a large variety of stressors. The scale is scored on a four-point basis, from 1 (not at all true) to 4 (exactly true). Higher scores represent higher self-efficacy [15].

### Statistics

Data are presented as the mean or median with 95% confidence interval (CI). To compare respondents with non-respondents, χ^2 ^and Student t tests were used for normally distributed data. The Student t test was used for comparisons of BDI, BHS, and GSE scores and one-way analysis of variance (ANOVA) was used to compare more than two independent groups. The level of significance was set at *P *< 0.05. In the analysis of BDI, BHS, and GSE scores, we accepted up to four missing items, and replaced them with the mean value. The data of sample characteristics are presented with valid percentages if cases had missing data. SPSS V.15.0 (SPSS Inc., Chicago, IL, USA) was used to analyze the data.

### Ethics

The study was performed in accordance with the Helsinki Declaration. Permission was obtained from The National Data Inspectorate and the Regional Ethics Committee. The patients gave informed verbal consent during the hospital stay to receive a questionnaire 3 months after the index hospital stay.

## Results

### Sample characteristics

The sample consisted of 242 patients. The mean age was 41 years (median, 37 years; range, 18 to 86 years). There were significantly more females (66%). Almost half of the participants lived alone. The vast majority were Norwegian citizens. A total of 30% were employed or students, 11% were on temporary sick leave, 10% were unemployed, 24% were permanently disabled, 10% were retired, and the remaining patients had other conditions, such as enrolment in military service or social welfare, or other/unknown situations. In all, 21% had completed only the minimum 9 years of primary and secondary school, 31% had completed high school, and 19% had completed college or university; this information for the remaining patients was unknown or missing.

Table 1 shows the comparison between this sample of respondents and the non-respondents. As shown, the respondents differed from the non-respondents, as the former included a significantly larger proportion of females and lower daily use of substances.

**Table 1:** Characteristics of patients treated for self-poisoning (respondents were compared with non-respondents)

Characteristics	Respondents (n = 242)	Non-respondents (n = 622)	*P *value
Mean age	40.8 years	40.4 years	0.746^a^
Females	66%	56%	0.007*
Norwegian	86%	86%	0.967
Living alone	42%	40%	0.605
Employed	19%	19%	0.887
Intention			0.382
Suicide attempt	42%	38%	
Appeal	27%	26%	
Substance-use-related poisoning	31%	36%	
Previous suicide attempt	44%	50%	0.087
Current psychiatric treatment	42%	40%	0.744
Previous psychiatric treatment	44%	50%	0.087
Daily use of substances	27%	39%	0.003*

^a^Student's t test was used; the other variables were compared using the χ^2 ^test.

*Significant at a 5% level.

### Reported given treatment

Six patients (2.5%) left the hospital against medical advice. Although only 14% of the remaining patients were registered in the hospital records as not being referred to follow-up assessment, 41% reported that they had not been offered referral before discharge (Table 2), that is, 77% of the patients who reported no referral were registered for follow-up assessment in the hospital. The discrepancy of reporting referrals was greatest among patients with suicide attempt and appeal.

**Table 2:** Referrals registered in hospital records versus self-reported offer of treatment at the time of discharge

	Suicide attempts	Appeals	Substance-use-related poisonings	Total
No referral registered in hospital records among non-respondents (n = 592)	6% (13/220)	10% (15/158)	37% (80/214)	18% (108/592)
No referral registered in hospital records among respondents (n = 220)	3% (3/93)	8% (5/60)	33% (22/67)	14% (30/220)
Not offered referral at the time of discharge according to self-report among respondents (n = 210)	28% (25/90)	39% (23/59)	61% (37/61)	41% (85/210)

Patients who left the hospital against medical advice, for whom intention was not registered hospital, or with missing answers were not included in this table.

Most patients were referred to psychiatric outpatient clinics (34%), psychiatric wards (23%), and GPs (23%). The corresponding figures, as reported by the patients, were 35, 13, and 18%. Among those who were registered for referral in the hospital charts but answered that no treatment was offered, referral to a general practitioner (28%) and psychiatric outpatient clinic (26%) was most common.

There was significantly less reporting of treatment offered at the time of discharge among the patients with a BDI score equal to or higher than 30 (n = 83), as 92% of them had a hospital record of referral versus 53% of patient-reported offers of referral. The corresponding figures among the group with a BDI score lower than 30 (n = 139) were 80% versus 64% (*P *= 0.01), indicating that the most depressed patients under-reported more often that follow-up was offered at the time of discharge (the six patients who left against medical device were excluded from this comparison).

In all, 23% of patients had their first follow-up appointment on the same day. Of these, 50% were admitted to a psychiatric ward. Furthermore, the first appointment was scheduled in less than 1 week for 27% and in less than 2 weeks for another 20% of patients. A waiting time for the first appointment of 3 weeks or longer was reported by 29% of patients. Nearly one-third (27%) of patients answered that their general practitioner was the most important professional to them during the 3-month period.

### Patient satisfaction

The overall satisfaction with different aspects of care in the general hospital was medium to rather satisfactory, with the highest scores observed for the general treatment in the hospital (3.9) and contact with the nurses (3.8), and lowest score registered for plans for follow-up at the time of discharge (2.9) (Table 3).

**Table 3:** Satisfaction with hospital care and health care services after discharge (1 = very dissatisfied; 5 = very satisfied)

	**n**^**a**^	Mean	95% CI
Hospital care			
General treatment	227	3.9	3.7 to 4.1
Contact with nurses	236	3.8	3.6 to 4.0
Contact with physicians	227	3.3	3.1 to 3.5
Consultation with social worker	94	3.3	3.0 to 3.6
Consultation with psychiatrist	140	3.0	2.8 to 3.3
Plans for follow-up at discharge	146	2.9	2.7 to 3.1
Health care services after discharge			
Psychiatric ward	84	3.6	3.3 to 3.8
Psychiatric outpatient clinic	42	3.3	2.8 to 3.7
General practitioner	41	3.4	2.9 to 3.9
Suicide prevention team	29	3.2	2.6 to 3.8
Psychiatric daycare	25	3.0	2.4 to 3.6
Private psychologist/psychiatrist	25	2.9	2.2 to 3.6
Outpatient substance use clinic	12	2.2	1.1 to 3.2
Family counseling/therapy	13	2.2	1.2 to 3.2

^a^The answer 'not relevant' was available for all categories, for example, not all patients received consultation with a social worker. The 'not relevant' alternative was not presented in this table, which was reflected in the number of respondents listed.

The general satisfaction with different follow-up services was moderate to rather satisfactory, with the exception of those admitted to a substance use clinic and to family counseling, which yielded lower satisfaction scores. The satisfaction with the psychiatric ward was rather good (3.6), and was best for the way they were met at admittance (3.4) and for the duration of the stay (3.3). The lowest scores were observed for psychotherapy and follow-up plans at discharge (both 2.9). The general satisfaction with the psychiatric outpatient clinic was above medium (3.3), with the highest scores observed for the way patients were met at admittance (3.9) and for psychotherapy (3.7), and the lowest scores registered for frequency of consultations and duration of treatment (both 3.5).

### Repeated self-harm

A total of 22% of patients reported new self-poisoning episodes after discharge, 49% reported no self-poisoning, and 29% did not answer this question. Among those who reported self-poisoning after discharge, 37% reported one episode and 31% reported two episodes during the past 3 months. Cutting was reported by 17% of the respondents, 40% of whom had cut themselves one or two times. Other potential harmful injuries were reported by 19% of patients. Among these patients, 38% reported one episode and 19% reported two episodes.

### Current psychiatric and drug misuse problems and self-perceived need for help

At the 3-month follow-up, 12% of the patients considered that their problems were so serious that they needed hospitalization, 31% needed help from a specialist, and 29% required help from a general practitioner (Table 4). Anxiety and depression were the most severe symptoms, but pain and alcohol and substance misuse were also present.

**Table 4:** Self-reported psychiatric and misuse problems and perceived need for help 3 months after hospitalization for self-poisoning (n = 242)

	No problems, not relevant, or missing data, n (%)	Some problems, do not need professional help, n (%)	Moderate problems, need help from general practitioner, n (%)	Rather serious problems, need help from specialist, n (%)	Very serious problems, need to be hospitalized into an institution, n (%)
Total^a ^(n = 242)	37 (15)	30 (12)	71 (29)	74 (31)	30 (12)
Anxiety	78 (32)	47 (19)	57 (24)	46 (19)	14 (6)
Depression	52 (22)	44 (18)	69 (29)	62 (26)	15 (6)
Pain	133 (55)	32 (13)	55 (23)	16 (7)	6 (3)
Eating disorders	161 (67)	42 (17)	19 (8)	14 (6)	6 (3)
Hallucinations	195 (81)	17 (7)	14 (6)	12 (5)	4 (2)
Paranoid ideation	190 (79)	15 (6)	20 (8)	13 (5)	4 (2)
Alcohol misuse	174 (72)	31 (13)	17 (7)	14 (6)	6 (3)
Substance misuse	181 (75)	24 (10)	22 (9)	9 (4)	6 (3)

^a^This row lists the highest score per patient for all categories.

### Psychiatric symptoms and self-efficacy

The mean score on the BDI was 23.3 (range, 0 to 54) for the whole group, which indicates moderate to severe depression. Females had significantly higher BDI scores than males (Table 5). Furthermore, those that were evaluated initially as suicide attempters had significantly higher depression scores than patients with substance-use-related poisoning.

**Table 5:** Self-reported Beck Depression Inventory, Beck Hopelessness Scale, and Generalized Self-Efficacy scale scores; differences according to suicide intention and gender 3 months after self-poisoning

	Total sample	Females	Males	Suicide attempt	Appeal	Substance-use-related poisoning
BDI 0 to 63 (95% CI)	23.3 (21.5 to 25.1)	25.2 (23 to 27.4)	20.3 (17.5 to 23.1; *P *= 0.009)	25.6 (22.9 to 28.3)	24.9 (21.8 to 28)	20.1 (16.7 to 23.4; *P *= 0.024)
BHS 0 to 20 (95% CI)	10.1 (9.7 to 11)	10.2 (9.2 to 11.2)	10.2 (8.7 to 11.6; *P *= 0.979)	11.1 (9.7 to 12.4)	10.6 (9.1 to 12.1)	8.7 (7.1 to 10.3; *P *= 0.064)
GSE 10 to 40 (95% CI)	25.2 (24.2 to 26.1)	25.2 (23.9 to 26.4)	25.5 (23.9 to 27.1; *P *= 0.745)	24.3 (22.6 to 26)	24.4 (22.7 to 26)	27.2 (25.4 to 28.9; *P *= 0.033)

One-way analysis of variance (ANOVA) was used to compare means regarding the intention variable, and an independent t test was used to analyze gender effect.

BDI = Beck Depression Inventory; BHS = Beck Hopelessness Scale; GSE = General Self-Efficacy Scale.

The average score on the BHS was 10.1 (range, 0 to 20), which indicates moderate hopelessness. There were no significant differences in hopelessness according to intention or gender.

The average GSE score was 25.2 (range, 10 to 30). Patients with substance-use-related poisoning scored significantly higher on the GSE than suicide attempters and appeals, whereas there were no significant differences according to gender.

## Discussion

The main findings of the present study were that many patients reported that no follow-up was offered, even though the hospital records show that they were registered with follow-up assessment. The satisfaction with treatment in the hospital and during follow-up was generally good. However, a significant proportion of the patients exhibited repeated self-harming behavior during the 3 months after discharge from the hospital and reported a considerably high level of psychiatric problems.

Many patients that had a hospital record of referral to follow-up reported that they had not been offered this assessment. These findings are in line with those of a Dutch study, in which 35% of suicide attempters did not remember whether aftercare arrangements had been made during their hospital stay when asked 7 days after discharge [17]. The fact that almost one-third of patients had to wait 3 weeks for their first appointment is one possible explanation for this result. During an acute crisis, patients may perceive such a long waiting time as no follow-up. Conversely, several patients who were definitely transferred directly to a psychiatric ward also reported absence of follow-up. Information about the referral may not have been understood or remembered by the patient, they may not have received the message in the hospital or written confirmation of their appointment.

Although the patients included in this study were rather satisfied with the treatment they received both in the hospital and after discharge, in the psychiatric outpatient clinic, they were less satisfied with the plans for follow-up and with the time from discharge to the first appointment. Taylor and coworkers reviewed attitudes towards clinical services among people engaging in self-harm. They found similar results, as the participants in their study also pointed out the need to improve access to care after discharge [11].

To date, there is no clear evidence of interventions or treatments that are effective after self-poisoning or injury [18]. However, because of the diversity and complexity of psychiatric problems, and their somatic and social character, it is important to provide coordinated, close, and systematic follow-up. Furthermore, a considerable number of patients drop out of treatment. Chain of care and early intervention after suicide attempts have yielded promising results, with lower rates of dropout from treatment and decreased number of readmissions [19]. In Norway, these kinds of services have been implemented in some parts of the country, to varying degrees; 50% of the hospitals report cooperation of follow-up [20]. As in many parts of the world, general practitioners are one of the cornerstones of the health care system. Almost one-third of the patients included in the present study considered that their general practitioner was most important in their care. This is supported by the results of another study [21], in which 64% of deliberate self-harm patients were satisfied with their general practitioner. Taken together, these findings suggest that follow-up performed by general practitioners may be helpful.

The results of the present study indicate that problems and a need for help were present 3 months after discharge, as a large group of patients felt that they needed help from their general practitioner or a specialist, or even hospitalization. The findings reported in a case-control study performed by Appleby and coworkers [22] indicate that a considerable number of the individuals who had completed suicide had their care reduced at the final appointment before they committed suicide. Thus, a correlation has been found between suicide risk and reduced level of care. Furthermore, the low scores observed on the GSE (25.2) show that the participating individuals' belief in their own ability to cope with novel or difficult situations and to deal with a large variety of stressors is low [23]. As a comparison, the mean GSE in the American general adult population is 29.5 [15].

The BHS scores obtained in our study were also high in the whole group (10.1). The mean BHS score in the general population of Ireland was 4.45 [16] versus 4.5 in the general population of Norway [24] and 10.2 among suicide attempters in a somatic hospital [25].

The depression score on the BDI observed in the present study was 23.3, which was elevated compared with that of suicide attempters in Bærum (19.4) [24]. In that study, a matched control group from the general population had a BDI score of 5.1. This indicates that the level of hopelessness and depression is much higher among patients with self-poisoning 3 months after discharge than in the general population. It should be noted that the study of Dieserud and coworkers, which we have used as a comparison for the sample of the present study, excluded drug-related overdoses. The comparison of the group classified as drug-related poisoning in the present study with the general population showed that the levels of depression and hopelessness were much higher in the former. Together with the considerable level of repetition of self-poisoning, cutting, and injuries reported, these results indicate a need for more active treatment strategies.

The assessment of intention is often performed in consultation or in accordance with the assessment of psychiatric personnel, and it is likely that the classification will influence decisions regarding follow-up plans at the time of discharge. Bjornaas and coworkers found that the agreement between the patients' self-reported intention and the physician's evaluation of intention, using the same categories as in the present study, was high [13]. In a 20-year follow-up study of all self-poisoning patients in Oslo, a suicidal motive upon admission were found to be the only predictor of later suicide as the risk were 3.1 times higher [2]. Considering the findings of the present study, this point must be emphasized further if the clinical evaluations are one of the main contributors to the choice of follow-up that is offered at the time of discharge, especially as the psychiatric symptoms were at similar levels in the appeal and suicide attempt groups 3 months after discharge.

### Strengths and limitations

This study was part of a multicenter study covering the capital of Norway and the largest surrounding municipality and therefore provides knowledge from one population with both self-poisoning and substance use related poisoning. It also provided the possibility of describing and comparing the respondents with the non-respondents regarding many variables. The use of validated and known instruments allowed the comparison of results. The knowledge about repeated acts based on self-reports from the patients who exhibited several repeated episodes of self-poisoning and cutting was another strength of the study, as many will not get in contact with health care services every time. It could be argued that there was a low response rate with regard to this question, but in spite of this, the amount of repeated self-harm and self-poisoning was considerable and clinically significant. Despite the rather low response rate obtained, the main conclusions of the study seem robust because even if the response rate were low, there is still a considerable group with high depression scores, long waiting times and further self-harming behavior.

Given the low response rate observed, the external validity of the present results may be questioned. The many similarities between respondents and non-respondents, however, support the validity of the main findings. A previous Swedish report indicated that the study of this patient group is difficult [26]. Nevertheless, almost one-third of the current patients answered the questionnaire. Unfortunately, we were not given permission from the Regional Ethics Committee to call the patients. In Norway, we have not until very recently had the opportunity to link patient data to register data, although verification of the results might have been useful and strengthened the findings. Previous research found that suicidal behavior, morbidity, and mortality are relatively high among people with no fixed place of abode [27]. It was impossible to avoid this selection bias, as postal addresses were not available. The term 'appeal' is controversial in the field of suicidology, as some fear the use of the term might implicate an underestimation of the seriousness of such acts. From clinical practice, there is a wide range between medically serious suicide attempts with an outspoken wish to die, and acts of self-poisonings which was never life threatening, but where the patient wished to communicate an unbearable situation to others in order to get help. In our study the main distinction between suicide attempters and appeals were their suicidal intent. Therefore, the terms 'moderate to high suicide intent' and 'low or no suicide intent' may have been used instead. We chose to keep the terms used in the original study forms used by the clinicians.

## Conclusions

There was a discrepancy between recorded plans for referral at discharge and the offer of treatment reported by the patients. Patients with substance-use-related poisoning were less likely to be referred to treatment; however, many suicide attempters also reported absence of follow-up. Many patients had to wait a long time before their first appointment. Although patients were generally satisfied with the treatment, they were less satisfied with plans for follow-up at discharge and with the waiting time. There was a considerable degree of depression, hopelessness, mental health problems, and repeated acts of self-harm 3 months after an episode of self-poisoning, indicating the need for more active follow-up measures. The group with the highest depression scores was less likely to remember that follow-up was planned at the hospital. Furthermore, the research and development of effective treatments for these patients should be emphasized.

## Competing interests

The authors declare that they have no competing interests.

## Authors' contributions

TKG analyzed the data and drafted the manuscript. MAB participated in the design, collection, and organization of data in the present part of the study and in the multicenter study. DJ conceived the multicenter study and supervised the work. GD coordinated the study and collection of data at the Bærum hospital. OE designed the study and supervised the statistical analyses and work. All authors participated in revising and improving the manuscript and approved the final version in accordance with the Vancouver statement.

## Authors' information

TKG is currently working as a PhD candidate in the Department of Acute Medicine at Oslo University Hospital. She is also cooperating with the Regional competence center about violence, traumatic stress and suicide prevention Eastern region of Norway. MAB is working in the Department of Acute Medicine at Oslo University Hospital. In her PhD thesis she focused on epidemiology, substance use, psychosocial factors and prognosis of self-poisonings in Oslo. DJ is the Director and Professor at the Department of Acute Medicine Oslo University Hospital. He has contributed to research in the field of clinical toxicology for more than 30 years. GD is a clinical psychologist and Director of the Department of Suicide Research and Prevention at the Norwegian Institute of Public Health, Division of Mental Health in Oslo, Norway. She has worked on suicide prevention in the municipality of Bærum, Norway, since 1984 and has been a suicide researcher at the Institute of Public Health since 1994. She is also working as advisor at Vestre Viken HF (Bærum hospital). OE is a consultant psychiatrist in the Department of Acute Medicine in Oslo University Hospital Ulleval. He is Professor at the Institute of Basic Medical Sciences, Department of Behavioral Sciences in Medicine, Faculty of Medicine, University of Oslo, Norway. He has been working with suicide prevention and research since 1979, and had a leading role in the development of a National Suicide Prevention program in Norway.
